# Vitamin D Levels in Chronic Rhinosinusitis in Patients With or Without Nasal Polyposis: A Systematic Review

**DOI:** 10.7759/cureus.46275

**Published:** 2023-09-30

**Authors:** Georgia Pantazidou, Ioannis Papaioannou, Charalampos Skoulakis, Efthimia Petinaki, Jiannis Hajiioannou

**Affiliations:** 1 Otolaryngology - Head and Neck Surgery, General Hospital of Patras, Patras, GRC; 2 Orthopedics, General Hospital of Patras, Patras, GRC; 3 Otolaryngology - Head and Neck Surgery, University Hospital of Larisa, Larisa, GRC; 4 Biopathology - Clinical Microbiology, University Hospital of Larisa, Larisa, GRC

**Keywords:** review article, chronic rhinosinusitis without nasal polyps, nasal polyps, chronic rhinosinusitis, vitamin d level

## Abstract

Chronic rhinosinusitis (CRS) is a large group of heterogeneous diseases characterized by extensive inflammation of the nasal mucosa and sinuses. Vitamin D (VD), as an immunoregulatory hormone, may play an important role in the pathophysiology of CRS. The purpose of this study is to review the existing literature that correlates VD levels with CRS with or without nasal polyps.

A systematic manual search was conducted in the PubMed and Google Scholar databases up to July 2023. Articles from PubMed and the first 100 articles from Google Scholar were recorded for our research. Keywords used were the following: vitamin D, chronic rhinosinusitis, and nasal polyps. Among the 134 articles retrieved, only 18 were eligible. The other 116 studies were excluded as they related VD levels with other conditions (e.g., allergic rhinitis) and for other reasons. However, we identified two more eligible records through the manual research of the above-mentioned 132 studies, and finally, 20 records were included in the current review. The review concerned case-control studies, prospective, retrospective, and cross-sectional studies.

Based on our review, we concluded that CRS patients are correlated with the lowest VD levels, accompanied by increased severity of the disease, especially in those with nasal polyposis. Patients can benefit from appropriate VD supplementation, and serum VD levels should be included in the laboratory assessment of CRS. However, due to the heterogeneity of the individuals involved, more well-designed clinical trials as well as randomized clinical trials should be conducted for further validation of the above findings in the general population in the future.

## Introduction and background

Chronic rhinosinusitis (CRS) includes a large spectrum of diseases with increased impact on individuals worldwide and is characterized by widespread inflammatory reactions of the nasal mucosa and paranasal sinuses. This condition is associated with the development and progression of lower airway diseases, including asthma [1]. It is one of the most common diseases, although there is no specific long-term treatment. The phenotypes of CRS are categorized as chronic rhinosinusitis with and without the development of nasal polyps (CRSwNP and CRSsNP, respectively) [2].

It is generally accepted that the fundamental action of vitamin D (VD) is to maintain the balance between calcium and phosphorus by regulating bone metabolism. Due to the expanding research focused on VD, many other biological functions have been revealed, while VD regulates hormone secretion and plays a key role in allergic diseases [3,4]. VD, as an immunoregulatory hormone, directly regulates a variety of cells, including monocytes, macrophages, epithelial cells, dendritic cells, and T cells. Although the exact mechanism of VD in immunoregulation remains unclear, recent studies suggest that VD may play an important role in the pathophysiology of CRS, which is characterized by chronic inflammation and occasionally allergic status.

This study aims to systematically evaluate the literature and highlight any possible correlation between VD levels and CRS development with all the above-mentioned phenotypes.

## Review

Systematic review

Eligible articles were defined according to Preferred Reporting Items for Systematic Reviews and Meta-Analyses (PRISMA) criteria.

Study design

The English language was mandatory for the included studies, investigating any possible correlation between VD levels and CRSwNP or CRSsNP. Eligible studies were the following: longitudinal prospective or retrospective studies, prospective cohort, case-control, cross-sectional, or randomized control studies, and those that reported the association of VD levels and CRS with or without nasal polyposis. The search was complemented by a hand scan of the reference lists of recognized articles. The search was confined to studies taken place in humans. The quality of the studies and risk of bias were assessed according to the Newcastle-Ottawa scale (Table 1), which is an eight-item checklist categorized into three sections: (1) selection of study groups, (2) compatibility of groups, and (3) outcome of interest.

**Table 1:** Quality of selected studies using the Newcastle-Ottawa scale.

Author	Selection	Compatibility and outcome	Total score
Schlosser et al. [5]	4	4	8
Erdag et al. [6]	4	4	8
Habibi et al. [7]	4	4	8
Mostafa et al. [8]	4	4	8
Baruah et al. [9]	4	3	7
Bavi et al. [10]	4	4	8
Hashemian et al. [11]	4	4	8
Wang et al. [12]	4	4	8
Konstantinidis et al. [13]	4	4	8
Wang et al. [14]	4	4	8
Tomaszewska et al. [15]	4	4	8
Thakur and Potluri [16]	4	3	7
Zand et al. [17]	4	4	8
Abdelaal et al. [18]	4	4	8
Shrestha et al. [19]	4	4	8
Shanaki et al. [20]	4	4	8
Lee et al. [21]	4	4	8
Abdel-Rahman et al. [22]	4	3	7
Lotfi et al. [23]	4	4	8
Ali et al. [24]	4	4	8

Exclusion criteria 

Reviews, meta-analyses, case series, case reports, animal studies, and studies published in languages other than English were excluded.

Types of participants and measurements

Patients with CRSwNP and CRSsNPS were eligible. The research was up to July 2023.

The included studies measured the following: serum 25-hydroxy-vitamin D (25(OH)VD) levels in patients with CRS in two groups (CRSwNP and CRSsNP), accompanied by a control group. Patients in the included studies were diagnosed with CRS based on clinical symptoms and/or computed tomography imaging, and they didn't receive any VD supplementation. The control group consisted of patients with no medical history concerning any otolaryngological disease. Articles that did not fulfill one or more of the above-mentioned inclusion criteria, duplicate studies, or studies that provided insufficient data were excluded.

Searching methodology

Manual, systematic research was done in PubMed and Google Scholar (first 100 articles). The research was restricted to July 2023. The keywords used were vitamin D, chronic rhinosinusitis, and nasal polyps. Among the 134 articles retrieved, only 18 were eligible. The other 116 studies were excluded as they related VD levels with other conditions (e.g., allergic rhinitis) and due to other reasons (e.g., written in languages other than English, literature reviews, meta-analyses, case reports, duplication). Although we identified two more eligible records through the manual research of the above-mentioned studies, 20 records were included in the current review [5-24].

We recorded the following: year of publication, country, author's name, type of study, and the total number of patients with gender specification. The authors of this systematic review have screened the titles and abstracts, while full texts were retrieved to identify any relevant studies mentioned in the reference list. All irrelevant articles were excluded, whereas full-text articles were checked by the authors to meet the inclusion criteria. In case of any controversy concerning the included articles, the issue was solved by consensus among the authors.

**Figure 1:**
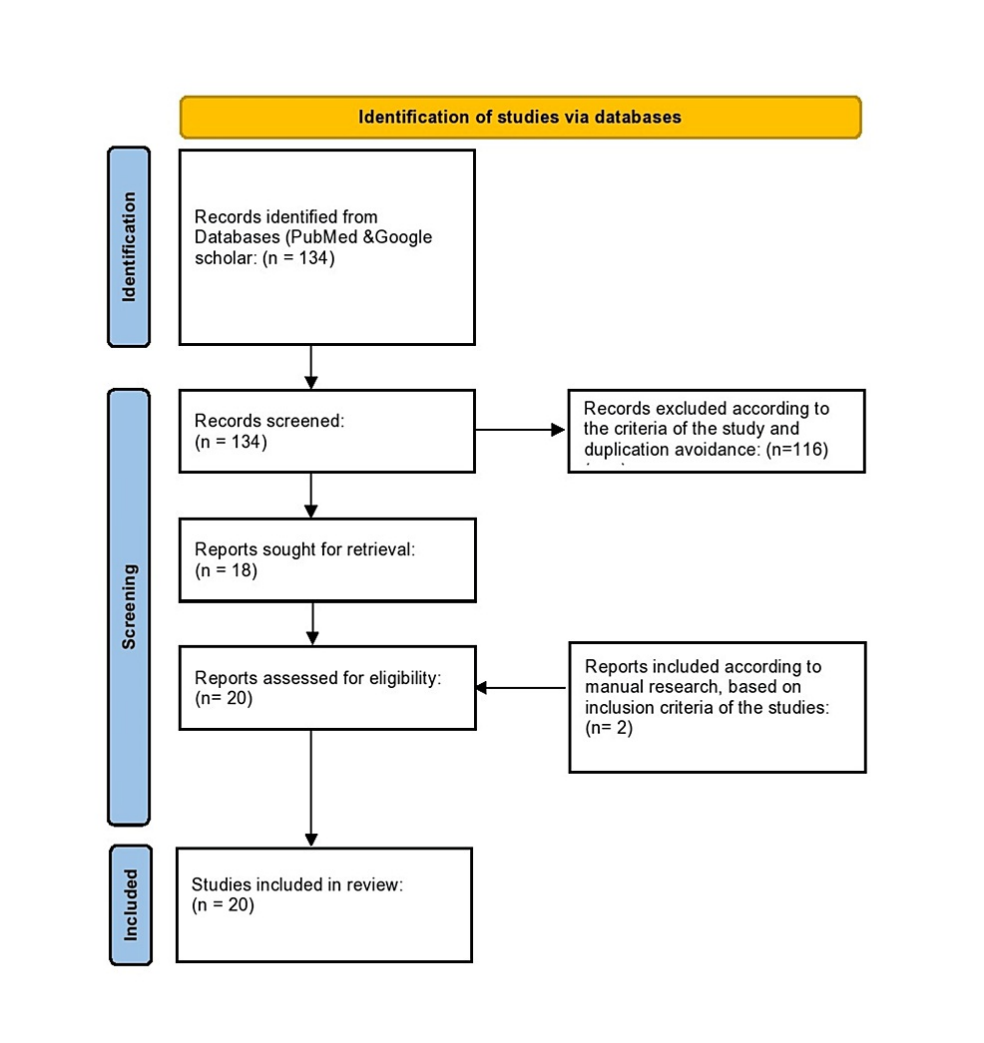
The PRISMA flow diagram.

The PRISMA flow diagram is demonstrated in Figure 1.

Study quality and risk of bias were assessed according to Newcastle-Ottawa (Table 1).

Results

A systematic manual search was conducted in PubMed, and the first 100 articles in the Google Scholar databases published up to July 2023 were selected. Using the terms vitamin D, chronic rhinosinusitis, and nasal polyps, a total of 134 studies were found. 116 of these were excluded as they concerned VD correlations with other conditions (e.g., allergic rhinitis) and for various other reasons (e.g., written in languages other than English, literature reviews, meta-analyses, and case reports). The remaining studies were 18. Although we identified two more eligible records through the manual research of the above-mentioned studies, finally 20 records were included in the current review.

According to the current literature, many studies have concluded that VD is actively involved in immunomodulation processes, causing anti-inflammatory and antioxidant effects in various immune-mediated pathologies. This review found that in CRSwNP or in CRSsNP, an immune-mediated pathogenic mechanism is recognized, in which VD plays a crucial role in improving both clinical and therapeutic outcomes. The purpose of this review is to highlight the effect of VD levels on the pathogenesis and clinical manifestations of CRSwNP/sNP and clarify any therapeutic value of this hormone, in addition to current therapies for CRS. For this review, we managed to collect 20 articles up to July 2023, matching the search criteria with the keywords VD levels, CRS, and nasal polyps [5-24].

Table 2 summarizes the correlations between CRS with or without NPs and VD levels.

**Table 2:** Correlations between chronic rhinosinusitis with or without nasal polyps and vitamin D.

Author	Year	Country	Study type	Patients-Gender	Results
Schlosser et al. [5]	2014	USA	Retrospective cohort study	70 patients (gender not reported)	CRSwNP patients are correlated with VD insufficiency or deficiency. African American patients are more vulnerable to this condition.
Erdag et al. [6]	2016	Turkey	Prospective study	55 male and 31 female	Expression of the VDR gene expression may be implicated with the pathophysiological mechanisms of nasal polyposis.
Habibi et al. [7]	2019	Iran	Cross-sectional control study	55 male and 62 female	Serum 25-OH-VD was significantly lower in CRS patients compared with that in non-CRS subjects.
Mostafa et al. [8]	2016	Egypt	Prospective case control study	37 male and 37 female	CRSwNP patients are correlated with significantly lower VD levels in comparison to CRSsNP patients and healthy subjects.
Baruah et al. [9]	2020	India	Retrospective case control study	76 male and 124 female	Higher prevalence of VD deficiency in CRS patients.
Bavi et al. [10]	2019	Iran	Cross-sectional case control study	191 male and 147 female	VD levels in CRSwNP group were significantly lower than in their healthy counterparts.
Hashemian et al. [11]	2020	Iran	triple-blind placebo-controlled clinical trial	28 male and 12 female	Nasal polyposis graveness was significantly lower in VD3 group in comparison to placebo group based on SNOT-22 score.
Wang et al. [12]	2019	China	Prospective case control study	46 male and 42 female	Serum 25-hydroxy VD3 levels are lower in Chinese CRSwNP patients.
Konstantinidis et al. [13]	2017	Greece	Prospective case control study	78 male and 74 female	Nasal polyps are strongly correlated with VD deficiency in patients with CRS.
Wang et al. [14]	2013	China	Prospective case control study	24 male and 14 female	Significantly lower VD level was found in a group of Taiwanese CRSwNP patients, which revealed an association with greater nasal polyp size.
Tomaszewska et al. [15]	2019	Poland	Prospective case control study	63 male and 103 female	VD, its receptor and its enzymes may play a role in CRS.
Thakur and Potluri [16]	2021	India	Prospective case control study	51 male and 40 female	Significantly lower VD level is associated with CRS, irrespective of presence or absence of NPs in adults residing at high altitudes.
Zand et al. [17]	2020	Iran	Prospective cross-sectional study	55 male and 38 female	Strong correlation was found between VD levels and severity of clinical manifestations in patients with CRSwNP.
Abdelaal et al [18]	2021	Egypt	Prospective cohort study	24 male and 18 female	VD3 has an effective way of lowering the severity of NPs and alleviating their manifestations.
Shrestha et al. [19]	2022	India	Prospective case control study	60 patients (gender not reported)	VD may play significant role in nasal polyposis formation.
Shanaki et al. [20]	2017	Iran	Retrospective case control study	44 male and 46 female	CRSwNP patients have significantly lower serum levels of 25-OH VD compared to healthy individuals.
Lee et al. [21]	2018	Korea	Retrospective case control study	13.177 male and 17.432 female	Low serum VD level might not be associated with increased prevalence of CRS in Korean adults; rather, patients with CRS showed higher serum VD levels than the control group.
Abdel-Rahman et al. [22]	2019	Egypt	Prospective case control study	24 male and 16 female	VD levels were significantly lower in patients with CRSwNP in comparison to control subjects.
Lotfi et al. [23]	2020	Iran	Prospective case control study	42 male and 18 female	Reversed relation between VD levels and proliferation of sinonasal fibroblasts.
Ali and Ali [24]	2023	Iraq	Observational study	40 male and 20 female	Patients suffering from CRSwNP had low VD levels compared to those with CRSsNP. VD deficiency correlated significantly with severity of CRSwNP.

Mostafa et al. measured VD serum levels in patients with allergic fungal rhinosinusitis (AFRS), CRS, and CRSwNP and concluded that VD levels in patients with CRSwNP and AFRS were significantly lower in comparison to those with CRSsNP and the control group. Thus, they suggested that the use of VD supplements might be a cheap and cost-effective therapy for the control of AFRS and CRSwNP, either alone or accompanied by the already existing therapies [8].

Abdel-Rahman et al. assessed VD levels in patients with nasal polyps, AFRS, CRS, and healthy controls. They concluded that VD may be a low-cost prophylactic and affordable option in the treating arsenal to reduce inflammation, either alone or in collaboration with traditional treatment options, in patients with nasal polyposis [22].

Konstantinidis et al. also conducted a prospective study in 152 adults to investigate if VD inadequacy is associated with the existence of nasal polyps in subjects with CRS, concluding that VD deficiency is correlated with polyp manifestation in CRS patients. More specifically, the lower the VD levels, the more severe the mucosal disease was found in imaging studies, accompanied by increased microbial colonization [13].

In the study by Lotfi et al., an inverse correlation was revealed between VD levels and nasal fibroblasts with tissue sampling, as the previous study reported. Thus, they concluded that VD has both therapeutic and prophylactic effects in CRSwNP [23].

The prospective case-control study of Wang et al. showed significantly lower VD levels in CRSwNP patients from Taiwan. The authors managed to correlate this VD deficiency with increased nasal polyp size in this particular group. The researchers concluded that serum VD levels could be added to the routine workup of patients suffering from CRS, and these data could be used to potentially help determine the disease severity [14].

The study by Bavi et al. revealed significantly lower VD levels in CRSwNP patients compared with their healthy counterparts. Disease severity was also inversely associated with serum VD levels. VD has now been shown to possess several immunological effects, especially on dendritic cells, T cells, and macrophages [10].

Schlosser et al., in their study, investigated the effect of VD inadequacy on the clinical symptoms of patients with CRSwNP, and they concluded that 55% of the patients did not have sufficient VD serum levels. Furthermore, low levels of VD were associated with more severe mucosal disease based on CT imaging, which is consistent with the findings of the present study [5].

Wang et al. concluded that 25-OH VD3 levels are lower in Chinese patients with CRSwNP and that preoperative serum VD values influence postoperative symptom improvement in a total of 88 subjects [12].

In a large epidemiological study by Jung Lee et al., a contrast to the previous studies emerged as the authors found that serum VD levels were higher in adult patients with CRS than in those without CRS in South Korea. According to this study, VD deficiency is not a risk factor for CRS, which contradicts the conclusions of all previous studies. This is the first epidemiological study conducted to investigate the relationship between VD and CRS and offers a substantial challenge to the previous findings of positive associations between low VD levels and CRS [21].

In the retrospective study by Baruah et al., a greater VD deficiency in CRS patients was revealed. The authors continued their study and separated the VD-deficient sample suffering from CRS into two subgroups. One subgroup received oral VD supplementation, and the other received a placebo. It was therefore shown that the additional administration of VD contributed to the relief of their symptoms [9].

In the prospective study by Abdelaal et al., 42 CRSwNP patients with VD deficiency (<30 ng/ml) were enrolled. The authors separated their sample into a VD supplementation group (22 subjects) and a placebo group (20 subjects). VD supplementation was shown to reduce the severity of nasal polyps in an effective manner, alleviating symptoms [18].

In the study by Hashemian et al., the VD-deficient patients with symptomatic CRS underwent FESS (functional endoscopic sinus surgery), and postoperatively, one group received 4000 IU oral VD supplementation and the other group received a placebo. In the study groups, there was an improvement in symptoms and endoscopy scores, although scores were reported to be significantly higher in the group of patients receiving VD supplementation in comparison to those in the placebo group [11].

A cross-sectional study by Faghih Habibi et al. confirmed that patients with CRSwNP or CRSsNP present significantly lower serum 25OH VD levels compared to subjects without CRS (control group) in 117 study subjects [7].

Another cross-sectional study by Zand et al. demonstrated the significant relationship between VD levels and disease severity in patients with CRSwNP [17]. Therefore, serum levels of VD could be added to the routine laboratory monitoring of patients with CRSwNP [17].

In the prospective study by Erdag et al., the authors assessed the relationship of VDR gene levels to VD levels in patients with CRSwNP, collecting tissue samples from nasal polyps and nasal mucosa samples from healthy subjects. It was concluded that VDR gene expression might be related to the pathogenesis or progression of nasal polyps [6].

In a subsequent prospective study by Tomaszewska et al., subjects were divided into three groups: CRSwNP, CRSsNP, and healthy individuals, and tissue from the middle nasal duct complex was also obtained to determine the levels of VDR and 1-α hydroxylase. A reduction in VDRs was found in CRS patients compared to healthy controls. Insignificant differences were observed in the expression of 1-α hydroxylase in the studied groups. Thus, it appeared that perhaps VD, its receptors, and its enzymes play an important role in CRS [15].

In a study by Shanaki et al., decreased serum 25-OH VD levels were found compared to healthy controls; however, VD binding protein levels were not different between healthy and CRS patients [20].

Also, in a prospective case-control study by Thakur and Potluri, low levels of VD were associated with CRS regardless of nasal polyp manifestation in individuals living at high altitudes. VD can independently predict CRS manifestation. Additionally, an inverse association of CRS severity with VD levels was confirmed [16].

The study performed by Shrestha et al. in January 2022 once again focused on the assessment of VD and interleukins 4, 5, and 13 levels in patients with CRSwNP. The study was prospective and found low levels of VD as well as interleukins 4, 5, and 13 in CRSwNP compared to healthy controls [19].

Finally, the most recent study by Ali et al. in April 2023 concluded that patients suffering from CRSwNP had low VD levels compared to those with CRSsNP. VD deficiency correlated significantly with the severity of CRSwNP. The estimated VD cutoff values from the current study could potentially be applied to evaluate the severity of the condition and the risk of nasal polyp growth [24].

Based on the above-mentioned studies, we concluded that low serum VD levels were significantly associated with CRS. Some studies that investigated the possible pathophysiological mechanisms of the nasal mucosa that correlate CRS with low serum VD levels have supported this evidence. Low VD levels can interfere with normal mechanisms to restrict mucosal inflammation and thus lead to outbreaks of CRS [25-27]. Additionally, an observational study by Wang et al. demonstrated the inhibitory effect of VD derivatives (calcitriol or tacalcitol) on the secretion of metalloproteinases MMP-2 and MMP-9, which reveals their potential application in patients with CRSwNP in Taiwan [28].

Furthermore, serum VD levels are known to be affected at random by such factors as race, sex, body mass index, geographic region, and season. A 2008 study found that VD levels in African American patients with CRS were significantly decreased compared to those in race- and sex-matched controls, whereas there was no major difference between whites using the same methodology [29]. It is therefore evident that race or dietary habits are important factors that can influence the serum VD levels of CRS subjects. A meta-analysis by Li et al. separated the studies into two subgroups, investigating the similarity of the involved studies. As shown, the group not living in the United States of America (USA) was observed to have a difference in VD levels between CRS and healthy [30], which may have been affected by other factors such as latitude or age of patients. For instance, studies have shown that people living in northern latitudes have always had low VD levels due to decreased exposure to the sun. In addition, aging has also been shown to affect serum VD levels [31]. The homogeneity of individuals is mandatory for future well-organized studies.

Discussion

VD deficiency is a worldwide public health issue. Obesity, lack of physical activity, and less time outdoors negatively affect its levels. It is known that origin and climate peculiarities affect VD levels. VD levels are typically higher in countries with more sunny days, although lifestyle modifications can mitigate this effect. Physical mechanisms such as angiogenesis, mucositis, and proliferation are closely connected with CRS, a malfunction due to low serum VD levels [30]. VD has anti-inflammatory actions, inhibits inflammatory cell proliferation, and acts as an immunomodulator agent. After initial hydroxylation in the liver, VD is transformed into the prohormone calcidiol (25(OH)D3), which is converted to calcitriol (1,25(OH)D3) by 1-α-hydroxylase mainly in the kidneys and other distal tissues. Then, it binds to the VD receptor in the cells (VDR), and many cell signaling pathways are activated [32]. Expression of proinflammatory cytokines (IL-6, IL-8, RANTES, and eotaxin) could be reduced in human nasal epithelial cells by 1,25(OH)VD, as shown in in vitro studies [33]. In addition, VD is essential for T-cell responses and T-regulatory cell responses to inflammation [34]. Based on the above-mentioned evidence, VD significantly influences the pathogenesis of CRS in combination with other agents. Due to the conflicting conclusions among existing studies, there is controversy concerning the relationship between VD levels and CRS, and till now, no consensus exists. To resolve this issue, well-designed and randomized controlled trials are paramount to establishing evidence-based medicine. More specifically, in the epidemiological study of a large sample by Lee et al. published in 2018, a significant contrast to the previously published studies was revealed, as the authors concluded that serum 25OH VD levels were higher in adult patients with CRS than those without CRS in South Korea. This epidemiological study contrasts with the majority of the current evidence concerning the relationship between VD levels and CRS and highlights that VD deficiency does not constitute a risk factor for CRS development [21]. It is worth noting that this is the only large study, which is contrary to the current evidence.

The prevalence of CRS is high, but its therapy is complicated in many cases because of the involvement of a variety of mechanisms in the etiology of the disease [35]. Presently, treatment of CRS relies on nasal glucocorticoid sprays in combination with saline (>200 mL) [36]. Current literature suggests that roughly 25-30% of CRS patients present with nasal polyposis. Histologically, the main category of cells found in the nasal polyps are eosinophils, and this type is known as “eosinophilic eCRSwNP.” CRSwNP occurs more often in Western countries [36]. The presence of eosinophils in the nasal polyps is correlated with the significant clinical severity of CRS, accompanied by an inadequate response to the administration of corticosteroids. Mucosal eosinophilia predisposes to CRS relapse, and current literature confirms the relationship between mucosal eosinophilia and postoperative recurrence [30]. It seems that eosinophils are useful biomarkers for CRS severity, possibly resulting immediately in the genesis of CRSwNP with T-helper type 2 (Th2) inflammation [37,38]. However, the current therapy with biological factors is successful in 50% of the patients. It is possible that patients will not respond to either pharmaceutical or surgical treatment in cases with nasal polyposis and co-existing asthma, aspirin-exacerbated respiratory disease (AERD), and AFRS [39]. Worse prognosis and greater disease severity are associated with lower VD blood levels. New biologic agents have recently been inserted in therapeutic guidelines, acting on specified targets, taking into account mostly the participation of Th2 type cells in the pathophysiology of CRSwNP and the great risk of failure of the existing protocols [40,41].

The effects of VD supplementation in patients with CRS have been analyzed by some authors, showing that uptake of 4000 IU of VD for a period of at least four weeks declines disease clinical manifestations. Adjunctive treatment with the dose mentioned before also decreases the incidence of nasal polyp relapses after FESS surgery. In patients with significant inflammation, first, high parameters of inflammation decreased and low VD increased after VD supplementation [17]. In this context, adjunctive treatment with VD, when levels are equal to the upper normal, can constitute an efficient and secure supplementary treatment to decelerate disease evolution in the worst types of CRSwNP. If this therapy fails, the next step is the administration of biologic factors and/or surgery. Dietary VD supplementation, even at the stage of a mild deficiency state, seems to have the ability to ameliorate the prognosis of allergic diseases such as atopic dermatitis, food allergies, and asthma [42].

A wealth of data concerning the connection between VD levels and the phenotypes of CRS, particularly CRSwNP, has been reported in this review. We also highlighted that low VD levels are correlated with greater disease severity and intense clinical manifestations. Therefore, VD supplementation incorporation into the treatment of patients with CRS could represent an evidence-based therapeutic strategy capable of adjuncting surgical and biological therapy to improve the clinical outcome and self-reported satisfaction of these patients. However, to confirm and establish the beneficial role of VD in the treatment of CRS, there is an absolute need for more well-designed prospective and randomized controlled clinical trials with a sufficient sample size.

## Conclusions

In summary, our review correlates lower levels of serum VD in patients with CRS and especially in CRSwNP subjects, indicating that people may benefit from appropriate VD supplementation. To further establish the association between VD3 and CRS, there is a requirement for longitudinal studies and/or randomized control trials. Ideally, future studies could use specific methods of measuring VD. Prospective studies, together with future randomized controlled trials, can hopefully help clarify the relationship between VD3 and CRS and determine the role of VD, if any, in providing adjunctive VD therapy in the treatment of CRS.

Based on the current evidence, we cannot definitively conclude if VD deficiency is a causative factor in CRS, while the disease severity is very difficult to quantify according to VD levels. Therefore, randomized clinical trials and prospective, well-designed studies should be accomplished in the future for the validation of these findings due to the heterogeneity of the participants. There is actually a need for further research to create guidelines for this type of therapy in CRS patients in addition to the main treatment.
